# SPOC (Small Private Online Course)-infused flipped classroom: a promising approach to address knowledge lag and practice gaps in infectious diseases teaching

**DOI:** 10.3389/fmed.2025.1702681

**Published:** 2025-12-16

**Authors:** Xiaohao Wang, Tiantian Li, Dazhi Zhang, Dachuan Cai, Hu Li, Shan Zhong

**Affiliations:** Key Laboratory of Molecular Biology for Infectious Diseases (Ministry of Education), Department of Infectious Diseases, Institute for Viral Hepatitis, The Second Affiliated Hospital of Chongqing Medical University, Chongqing, China

**Keywords:** SPOC, flipped classroom, infectious diseases, medical education, randomized controlled trial

## Abstract

**Objective:**

This study aimed to design and implement a “SPOC (Small Private Online Course) + flipped classroom” teaching model for Infectious Diseases, and to evaluate its effectiveness in improving students’ academic performance, clinical practical ability, and teaching satisfaction, so as to provide a scientific practical reference for the teaching reform of Infectious Diseases.

**Methods:**

A total of 361 five-year clinical medicine students (2018 and 2019 grades) from Chongqing Medical University were selected as the research objects. They were randomly divided into an experimental group (*n* = 61) receiving SPOC-blended flipped classroom teaching and a control group (*n* = 300) receiving traditional Lecture-Based Learning (LBL) teaching. There were no significant differences in baseline data between the two groups (*P* > 0.05), indicating comparability. The two groups had the same teaching content, teaching hours, and teaching staff. After the course, the academic performance [final theoretical examination and Objective Structured Clinical Examination (OSCE)] and teaching satisfaction of the two groups were compared.

**Results:**

The scores of the final theoretical examination (81.7 ± 6.64 vs. 77.2 ± 6.13, *P* < 0.001) and OSCE (83.8 ± 4.53 vs. 79.9 ± 6.29, *P* < 0.001) in the experimental group were significantly higher than those in the control group. In terms of teaching satisfaction, the scores of the experimental group in all dimensions, including “practicality of teaching content” (4.4 ± 0.5 vs. 3.1 ± 0.6, *P* < 0.001), “attractiveness of teaching methods” (4.6 ± 0.4 vs. 3.0 ± 0.7, *P* < 0.001), “effect of ability improvement” (4.5 ± 0.5 vs. 3.2 ± 0.6, *P* < 0.001), and the total score (4.5 ± 0.4 vs. 3.2 ± 0.6, *P* < 0.001), were significantly higher than those in the control group.

**Conclusion:**

The “SPOC + flipped classroom” teaching model can effectively improve the academic performance, clinical practical ability, and teaching satisfaction of clinical medicine students in Infectious Diseases learning. It provides a feasible practical path for the teaching reform of Infectious Diseases and a replicable paradigm for mixed teaching in other medical courses.

## 1 Introduction

Infectious Diseases is a core course for clinical medicine majors, integrating both theoretical and practical attributes. Its knowledge system is characterized by a rapid update rate (such as the discovery of new coronavirus variants and the iteration of diagnostic and therapeutic guidelines for infectious diseases) and a close connection with the public health field (such as emergency prevention and control strategies for public health emergencies). The traditional Lecture-Based Learning (LBL) teaching model, which is teacher-centered and adopts a “cramming” knowledge transfer method, has inherent limitations of “emphasizing theoretical indoctrination over practical skill training” and “prioritizing knowledge memorization over clinical thinking improvement” (1, 2). Under this model, students are mostly in a passive state of knowledge acceptance, making it difficult to effectively combine abstract theoretical knowledge with complex clinical cases. When facing real diagnosis and treatment scenarios, they often show shortcomings in independent problem-analysis and problem-solving abilities, which are insufficient to meet the needs of modern medical education for cultivating high-quality clinical talents (3).

With the advent of the Education Informatization 2.0 era, Small Private Online Course (SPOC) has become an important link connecting online independent learning and offline classroom teaching, relying on its unique advantages of “targeted resource push, controllable learning scale, and personalized learning support” (4, 5). The flipped classroom, through the reconstruction of the teaching process of “knowledge transfer before class, knowledge internalization in class, and knowledge expansion after class”, returns the initiative of learning to students, effectively stimulating their initiative and creativity in learning (6, 7). Recent studies have highlighted the efficacy of SPOC in enhancing learning engagement and knowledge retention in medical education. By offering targeted resources and personalized feedback, SPOC addresses the limitations of one-size-fits-all approaches (8). The flipped classroom model, when combined with SPOC, has demonstrated improved student performance in clinical skill acquisition and critical thinking (9). This integrated approach has been particularly effective in disciplines requiring rapid knowledge updates, such as infectious diseases (10). The “SPOC + flipped classroom” blended teaching model formed by the organic integration of the two has been applied in the teaching of multiple medical courses such as Physiology, Surgery, and Internal Medicine, and has been proven to significantly improve teaching effectiveness (11–13). However, in the field of Infectious Diseases, the systematic application research of this model is still relatively weak, especially the lack of teaching program design and empirical research targeting the “theory-practice-public health” three - dimensional integration characteristics of Infectious Diseases (14).

Based on this, this study took students majoring in clinical medicine at Chongqing Medical University as the research objects, designed and implemented the “SPOC + flipped classroom” teaching program for Infectious Diseases, with core chapters such as “viral hepatitis,” “AIDS,” and “novel coronavirus infection” as the practical carriers. A randomized controlled trial was conducted to verify the teaching effectiveness of this model, aiming to provide a scientific practical reference for the teaching reform of Infectious Diseases and help cultivate high - quality medical talents with a solid theoretical foundation, excellent clinical skills, and good public health literacy.

## 2 Materials and methods

### 2.1 Study participants

Five-year clinical medicine students of grades 2018 and 2019 from Chongqing Medical University were selected as the research objects. The inclusion criteria were as follows: (1) Having completed the study of basic medicine courses and preliminary clinical medicine courses, and having the knowledge foundation for conducting Infectious Diseases learning; (2) Having registered for the “Infectious Diseases” course normally and having a complete course learning cycle; (3) Voluntarily participating in this study. The exclusion criteria were: (1) International students (due to differences in teaching language and evaluation standards, it was difficult to ensure the consistency of the study); (2) Students who were absent for more than 3 times during the course learning period (which might affect the integrity of the teaching intervention and lead to data bias); (3) Students who refused to participate in the teaching effectiveness evaluation or whose questionnaire answers were incomplete. Finally, a total of 361 students were included. They were divided into an experimental group (61 students) and a control group (300 students) at a ratio of 1:5 using a random number table. The differences in the admission scores (total college entrance examination scores, average scores of basic medicine courses) between the two groups were not statistically significant (*P* > 0.05), indicating comparability.

### 2.2 Intervention programs

The teaching content of the two groups was strictly set in accordance with the “National Undergraduate Education and Teaching Guidelines for Clinical Medicine Majors in Colleges and Universities (2021 Edition)” and the “Infectious Diseases Teaching Syllabus” of Chongqing Medical University, covering 20 core chapters such as “viral hepatitis,” “hemorrhagic fever with renal syndrome,” “AIDS,” “cholera,” and “novel coronavirus infection.” The “Infectious Diseases” (9th edition, edited by Li Lanjuan and Ren Hong) published by People’s Medical Publishing House was used as the unified textbook. The lecturers were all teachers from the Infectious Diseases Teaching and Research Office of the Second Clinical College of Chongqing Medical University, with more than 5 years of clinical and teaching experience in Infectious Diseases. There was no significant difference in the teaching level of the teachers between the two groups as evaluated by peers. The total course hours were 37, including 28 h of theoretical courses and 9 h of clinical demonstration courses. The same set of theoretical examination papers and OSCE assessment standards were used at the end of the term to ensure the fairness and consistency of the evaluation.

#### 2.2.1 Experimental group: SPOC-blended flipped classroom teaching

**FIGURE 1:**
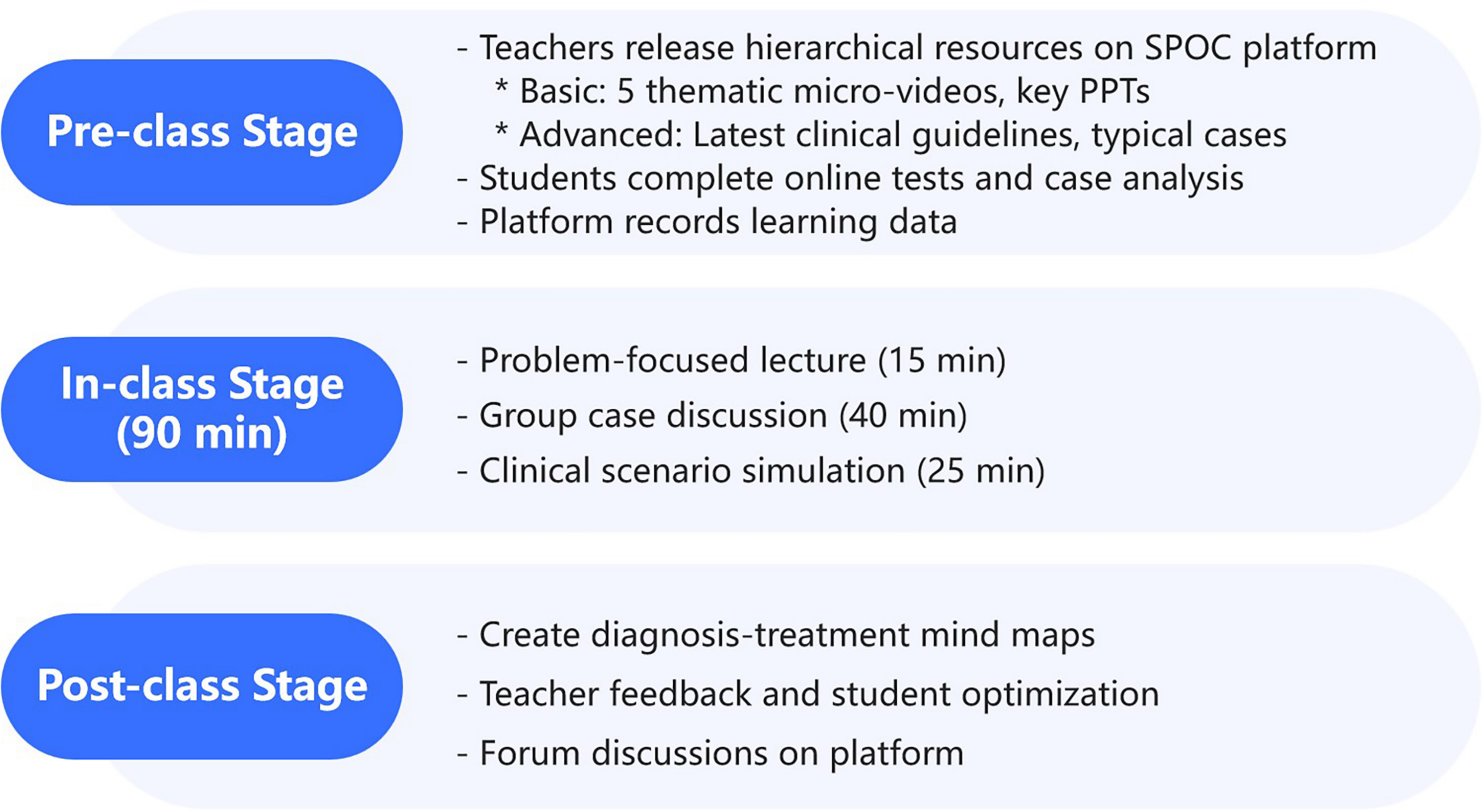
SPOC + flipped classroom teaching model.

A three-stage closed-loop teaching model of “online independent learning before class-offline in-depth internalization in class-online expansion and consolidation after class” was adopted (Figure 1). Taking the chapter of “viral hepatitis” as an example, the specific implementation process was as follows:

Pre-class Stage (1 week): Teachers released hierarchical teaching resources and personalized learning tasks relying on the SPOC teaching platform of Chongqing Medical University (built based on the Blackboard open-source system). ① Basic resources: 5 thematic micro-videos (each 8–12 min long, recorded around core knowledge points such as “etiological characteristics of various hepatitis viruses,” “transmission routes and epidemiological characteristics,” “clinical manifestations and classification,” “interpretation of laboratory tests,” and “basic principles of treatment”). The videos were co-produced by chief physicians of the Infectious Diseases Department and educational technology professionals to ensure the professionalism of the content and the intuitiveness of the presentation; PPT of key content of the textbook (marking knowledge key and difficult points and learning objectives). ② Advanced resources: PDF version of the “Guidelines for the Prevention and Treatment of Viral Hepatitis (2022 Edition)” (jointly released by the Hepatology Branch and the Infectious Diseases Branch of the Chinese Medical Association), with teachers marking clinical key contents such as antiviral treatment indications and liver failure rescue procedures in advance; 3 cases of typical clinical case data (selected from real cases of the Second Clinical College of Chongqing Medical University, desensitized, including complete clinical information such as patient medical history, physical examination results, liver function test sheets, virus marker detection reports, and imaging examination images). ③ Learning tasks: Online tests (15 objective questions covering basic knowledge points, with the platform automatically correcting and providing immediate feedback on answer explanations); Case analysis assignments (requiring students to submit a preliminary diagnosis, differential diagnosis ideas, and a framework of treatment plans based on the provided case data. Teachers released case analysis writing guidelines on the platform in advance). Students could arrange their learning time independently according to their own learning rhythm. The platform automatically recorded learning data (including video viewing completion rate, test accuracy rate, and assignment submission time). Teachers could grasp the students’ learning progress and weak links in real - time through the platform data.

In-class Stage (90 min): ① Focused explanation of problems (15 min): Based on the pre-class platform learning data, teachers focused on knowledge points with a high error rate among students (such as “interpretation of hepatitis B five serological markers,” “key points of differential diagnosis of different types of hepatitis,” and “principles of antiviral drug selection”), and gave targeted explanations combined with typical clinical cases to strengthen knowledge understanding. ② Case discussion (40 min): Students were divided into groups of 6–7 people. Each group was assigned a complex clinical case (such as “hepatitis B cirrhosis complicated with liver cancer,” “severe hepatitis E in pregnant women,” and “chronic hepatitis C complicated with autoimmune liver disease”). Within the group, members divided the work to complete the tasks of “medical history sorting-diagnosis derivation-treatment plan formulation-prognosis evaluation.” Then, each group selected one representative to give an 8-minute report. Other groups raised questions and made comments from the perspectives of “completeness of diagnostic basis,” “rationality of treatment plan,” and “logicality of clinical thinking.” Finally, teachers summarized and induced to guide students to form a systematic clinical thinking. To foster a supportive atmosphere for mistake tolerance and inclusive participation: Teachers established ‘no-criticism’ discussion rules (e.g., “focus on ideas, not personal opinions”) to reduce students’ fear of errors; Group roles (e.g., note-taker, presenter, questioner) were rotated weekly to ensure shy students had structured opportunities to participate; Teachers proactively invited quiet group members to share supplementary points after formal presentations, encouraging leadership development through gradual engagement. ③ Practical simulation (25 min): “Doctor-patient” role-playing practice was carried out, setting up scenarios such as “communication on antiviral treatment plans for hepatitis B patients,” “screening and health education guidance for close contacts of hepatitis A patients,” and “health education on complication prevention for patients with post-hepatitis cirrhosis.” Students played the roles of doctors and patients respectively. Teachers gave on-site comments from the perspectives of “doctor-patient communication skills,” “accuracy of professional knowledge expression,” and “reflection of humanistic care” to improve students’ clinical communication skills.

Post-class Stage (1 week): ① Expansion tasks: Students were required to draw a “viral hepatitis diagnosis and treatment process mind map” in groups (which should include the whole process of diagnosis, treatment, and follow-up). ② Feedback and optimization: Teachers completed the correction of students’ assignments within 3 working days. For common problems (such as “missing key links in the diagnosis and treatment process” and “imbalance between professionalism and popularity of popular science content”), supplementary explanation videos or documents were released on the platform. Students revised and improved their assignments according to the teacher’s feedback and submitted them again. At the same time, students were encouraged to share learning experiences and questions in the platform forum, and teachers and classmates participated in the discussion together to form a mutual learning atmosphere.

#### 2.2.2 Control group: traditional LBL teaching

**FIGURE 2:**
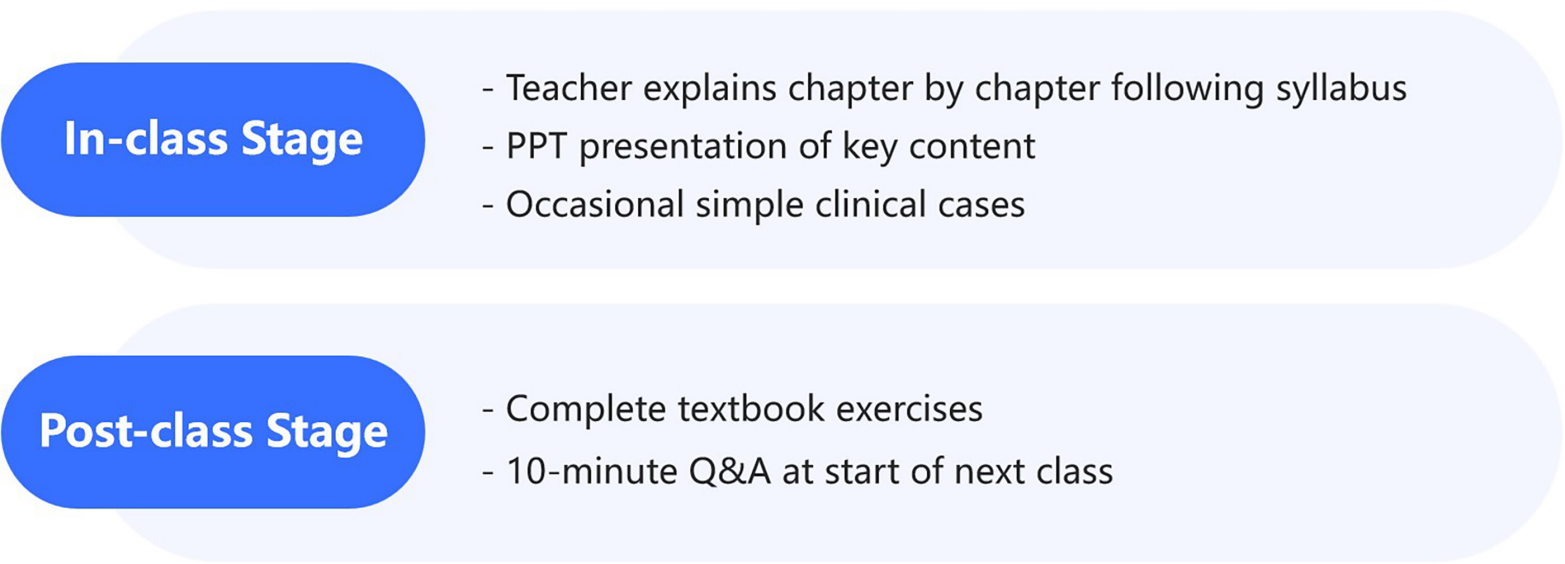
Traditional LBL teaching model.

The traditional teaching model of “teacher’s classroom teaching + students’ independent review after class” was adopted (Figure 2). Teachers explained the theoretical knowledge chapter by chapter in accordance with the teaching syllabus, and showed key contents (such as hepatitis virus structure diagrams, clinical staging timelines, and lists of therapeutic drugs) combined with PPT, occasionally interspersing simple clinical case examples. After class, after-class exercises in the textbook (10–15 questions per chapter) were assigned. Students completed the review and exercise answers independently. At the beginning of the next class, teachers spent 10 min answering the questions existing in the students’ assignments in a centralized manner, and no special case discussions or practical simulation activities were carried out.

### 2.3 Teaching implementation guarantee

Resource Guarantee: A teaching resource development team was established, consisting of clinical physicians from the Infectious Diseases Department (3 chief physicians, 5 associate chief physicians), medical education experts (2 professors of education), and educational technology personnel (2 persons). They jointly completed the production and review of resources such as micro-videos, case data, and guideline interpretations on the SPOC platform to ensure the quality of resources. The case data were all selected from real cases of the Second Clinical College of Chongqing Medical University in the past 3 years. After being approved by the hospital ethics committee, they were desensitized to avoid privacy leakage.

Teacher Training: Before the implementation of teaching, two special training sessions on flipped classroom teaching skills were conducted for the lecturers. The content included “methods of organizing case discussions,” “strategies for guiding role-playing,” “creating an interactive classroom atmosphere,” and “interpretation and application of SPOC platform data.” After the training, a simulated teaching assessment was conducted to ensure that teachers were proficient in mastering the flipped classroom teaching process and skills and could effectively control the classroom rhythm.

Technical Support: Two full-time technical personnel were arranged to be responsible for the daily operation and maintenance of the SPOC platform. They tested the functions of the platform such as video playback, assignment submission, and online tests 1 day before each round of classes. During the class, they monitored the operation status of the platform in real - time and promptly solved technical problems such as stuttering and abnormal data loading encountered by students. In the first week of the new semester, a SPOC platform operation training was conducted for the students in the experimental group. Through on-site demonstrations and practical exercises, all students were ensured to be proficient in mastering operations such as resource viewing, task submission, and forum interaction.

### 2.4 Evaluation indicators and data processing

#### 2.4.1 Academic performance evaluation

Final Theoretical Examination: A closed-book examination with a 100-point system was adopted. The test paper was jointly proposed by 2 senior professors from the Infectious Diseases Teaching and Research Office of Chongqing Medical University and used after being reviewed and approved by external peer experts. The structure of the test paper included: multiple-choice questions (40 points, testing the memory and understanding of basic knowledge points), short-answer questions (30 points, testing the ability to sort out and summarize the knowledge system), and case analysis questions (30 points, testing the ability to apply knowledge and clinical thinking). The examination time was 120 min. The marking was conducted uniformly by non-lecturers using a streamlined operation method to ensure the consistency of the scoring standards.

Objective Structured Clinical Examination (OSCE): A 100-point system was adopted. Referring to the “Chinese Physician Qualification Examination Practical Skills Examination Syllabus,” 3 examination stations were set up, with 15 min of examination time at each station. ① Medical history collection station: Standardized patients (medical students who had received professional training) were provided, and students were required to complete the collection of medical history related to “viral hepatitis” within the specified time. ② Physical examination station: Students were required to conduct abdominal physical examination (including inspection, palpation, percussion, and auscultation) on patients with “hepatitis cirrhosis” and describe the examination results correctly. ③ Case analysis station: A case of complex hepatitis was provided, and students were required to complete the diagnosis, differential diagnosis, and formulation of treatment plans on - site and report the ideas orally. At each station, 2 teachers with the title of associate chief physician or above in the Infectious Diseases Department who were unaware of the grouping scored independently. The average score was taken as the final OSCE score of the students. The scoring standards were formulated in advance and uniformly trained to ensure the fairness of the assessment.

#### 2.4.2 Teaching satisfaction evaluation

A self-designed “Infectious Diseases Teaching Satisfaction Questionnaire” (Supplementary Table 1) was developed by adapting the validated “Teaching Satisfaction Questionnaire” (15) and further modifying its items to align with the disciplinary characteristics of Infectious Diseases teaching (e.g., integrating content related to clinical case analysis and public health practice of infectious diseases). The questionnaire was designed based on a 5-point Likert scale (1 = very dissatisfied, 2 = dissatisfied, 3 = average, 4 = satisfied, 5 = very satisfied). It included 5 dimensions and 15 questions: “applicability of teaching resources,” “effectiveness of classroom teaching,” “after-class learning support,” “improvement of learning initiative and ability,” and “overall teaching effect.” The content validity of the questionnaire was reviewed by 2 experts from the Medical Education Research Institute of Chongqing Medical University. The reliability was tested through a pre-survey (*n* = 50), and the results showed that the Cronbach’sαcoefficient was 0.83, indicating good reliability and validity of the questionnaire. Within 1 week after the end of the course, the questionnaires were distributed to the students of the two groups through the Wenjuanxing platform, and filled in anonymously. A total of 361 questionnaires were distributed, and 361 valid questionnaires were recovered, with an effective recovery rate of 100%.

Preliminary Teacher Feedback: After the course, 8 participating teachers provided brief feedback on their adaptability to the new model, with 6 teachers (75%) reporting ‘basic proficiency in guiding case discussions’ but 4 teachers (50%) mentioning ‘higher time investment in pre-class resource preparation and post-class feedback’ compared to traditional teaching. This preliminary feedback indicates the need for more systematic teacher experience investigation in subsequent studies.

#### 2.4.3 Data processing

SPSS 26.0 statistical software was used for data analysis. Measurement data were expressed as mean ± standard deviation ($\bar{\text{x}}$ ± s), and independent sample *t*-test was used for comparison between groups; Count data were expressed as the number of cases (rate), and χ^2^ test was used for comparison between groups. A *P*-value of <0.05 was considered statistically significant.

### 2.5 Ethical approval

This study was approved by the Ethics Review Committee of the Second Clinical College of Chongqing Medical University, and strictly followed the ethical principles of the Declaration of Helsinki. Before the study, all students were informed of the research purpose, methods, risks, and benefits. During the research process, students’ privacy was strictly protected, and all data were only used for the analysis of this study to ensure the compliance of research ethics.

## 3 Results

### 3.1 Comparison of academic performance between the two groups

The scores of the final theoretical examination and OSCE in the experimental group were significantly higher than those in the control group, and the differences were statistically significant (both *P* < 0.001), as shown in Table 1.

**TABLE 1:** Comparison of academic performance between the two groups ($\bar{\text{x}}$ ± s, scores).

Group	Number of case	Final theoretical examination	OSCE
Experimental group	61	81.7 ± 6.64	83.8 ± 4.53
Control group	300	77.2 ± 6.13	79.9 ± 6.29
*t*-value	–	5.153	4.604
*P*-value	–	<0.001	<0.001

*t*-value: A statistical test indicator reflecting the magnitude of the mean difference between the two groups; a larger absolute t-value indicates a greater difference in scores between groups. *P*-value < 0.05 is considered statistically significant, and *P* < 0.001 indicates an extremely significant difference (key indicator for judging result reliability.

### 3.2 Comparison of teaching satisfaction between the two groups

The experimental group (SPOC + flipped classroom) achieved significantly higher scores than the control group (traditional LBL) in all five satisfaction dimensions and the total score, with statistically significant differences (all *P* < 0.001), as shown in Table 2. The total satisfaction score of the experimental group was 4.47 ± 0.38, which was 1.29 points higher than that of the control group (3.18 ± 0.52). Among the five dimensions, the experimental group showed the most prominent advantage in “Learning Initiative and Competence Improvement” (experimental group: 4.53 ± 0.41 vs. control group: 3.15 ± 0.63), followed by “Classroom Teaching Effectiveness” (experimental group: 4.48 ± 0.39 vs. control group: 3.09 ± 0.68).

**TABLE 2:** Comparison of teaching satisfaction between the two groups ($\bar{\text{x}}$ ± s, scores).

Satisfaction dimension	Survey content	Experimental group (*n* = 61)	Control group (*n* = 300)	*P*-value
Applicability of teaching resources	1. Teaching resources can clearly explain core knowledge of Infectious Diseases	4.41 ± 0.49	3.08 ± 0.61	<0.001
	2. Teaching resources help verify mastery of knowledge points	4.34 ± 0.52	3.13 ± 0.60	<0.001
	3. Teaching resource content matches learning objectives of core chapters	4.32 ± 0.51	3.15 ± 0.59	<0.001
	Dimension subtotal	4.37 ± 0.45	3.12 ± 0.58	<0.001
Effectiveness of classroom teaching	4. Classroom teaching effectively solves doubts in learning	4.35 ± 0.50	3.10 ± 0.66	<0.001
	5. Classroom activities help connect theoretical knowledge with clinical practice	4.57 ± 0.43	3.05 ± 0.71	<0.001
	6. Class time allocation is reasonable and does not affect learning efficiency	4.38 ± 0.48	3.12 ± 0.65	<0.001
	Dimension subtotal	4.48 ± 0.39	3.09 ± 0.68	<0.001
After-class learning support	7. After-class tasks help deepen understanding of Infectious Diseases knowledge	4.28 ± 0.54	3.08 ± 0.63	<0.001
	8. Teacher feedback on after-class tasks is timely and guiding	4.45 ± 0.49	3.01 ± 0.67	<0.001
	9. After-class learning support helps adjust independent learning rhythm	4.21 ± 0.53	3.13 ± 0.62	<0.001
	Dimension subtotal	4.32 ± 0.47	3.07 ± 0.64	<0.001
Learning initiative and competence improvement	10. Current teaching model stimulates learning initiative for Infectious Diseases	4.62 ± 0.40	3.04 ± 0.72	<0.001
	11. Mastery of theoretical knowledge is more solid through the current model	4.49 ± 0.46	3.18 ± 0.61	<0.001
	12. Current teaching model helps improve core competencies	4.48 ± 0.45	3.18 ± 0.60	<0.001
	Dimension subtotal	4.53 ± 0.41	3.15 ± 0.63	<0.001
Overall teaching effect	13. Core chapter content covers meet course learning objectives	4.43 ± 0.48	3.20 ± 0.59	<0.001
	14. Curriculum arrangement matches the teaching model to achieve teaching goals	4.55 ± 0.42	3.16 ± 0.63	<0.001
	15. Recognize the application value of the current model in Infectious Diseases teaching	4.46 ± 0.47	3.23 ± 0.58	<0.001
	Dimension subtotal	4.49 ± 0.36	3.21 ± 0.59	<0.001
Total satisfaction score		4.47 ± 0.38	3.18 ± 0.52	<0.001

## 4 Discussion

### 4.1 Core mechanisms of SPOC + flipped classroom in improving teaching effectiveness

#### 4.1.1 Precision of knowledge transfer to meet individualized learning needs

The hierarchical teaching resource design (basic resources and advanced resources) of the SPOC platform can specifically meet the learning needs of students with different foundations. Students with weak foundations can consolidate theoretical knowledge through basic resources such as micro-videos and key PPTs, avoiding the impact of knowledge gaps on learning effects; students with strong learning abilities can expand their clinical horizons through advanced resources such as diagnosis and treatment guidelines and complex cases, and deeply understand the application of theoretical knowledge in clinical practice (16, 17). This effectively solves the problems of “insufficient learning” and “excessive learning pressure” caused by the “unified progress and one-size-fits-all” approach in traditional LBL teaching (18). At the same time, the real-time learning data recorded by the platform (such as video viewing progress and test error rate) enables teachers to accurately identify students’ knowledge weaknesses and conduct targeted in-depth explanations in class, thereby improving teaching efficiency (19).

#### 4.1.2 Scenario-based ability training to strengthen clinical practical literacy

The in-class case discussion and role-playing sessions transform the abstract theoretical knowledge of Infectious Diseases into real clinical practice scenarios. In the discussion of complex cases, students gradually establish systematic clinical thinking through the complete process of “sorting out medical history-deriving diagnosis-formulating plans”; in the doctor-patient communication role-playing, students experience clinical communication scenarios firsthand, which not only improves their ability to apply professional knowledge but also cultivates their awareness of humanistic care and communication skills (20–23). This scenario-based teaching model is highly consistent with the core goal of the OSCE assessment, which directly leads to a significant improvement in the OSCE scores of the experimental group (experimental group: 83.8 ± 4.53 vs. control group: 79.9 ± 6.29, *P* < 0.001), confirming the value of this model in cultivating clinical practical ability.

#### 4.1.3 Activation of learning participation to stimulate intrinsic learning Motivation

The “SPOC + flipped classroom” model, through the closed-loop design of “independent pre-class learning-in-class interactive discussion-post-class expansion and feedback,” has completely changed the “teacher-led and student-passive” situation in traditional LBL teaching (10, 20, 21). The ability to independently arrange pre-class learning time and choose the order of resource learning gives students the autonomy in learning (24); the in-class group collaborative discussions and role-playing presentations provide students with a platform for expression and communication, enhancing their sense of participation in learning (25); the post-class personalized feedback and forum interactions allow students to obtain timely evaluations of their learning effects, strengthening their sense of learning achievement (26). The teaching satisfaction data show that the score of the experimental group in the “attractiveness of teaching methods” dimension (4.6 ± 0.4) is significantly higher than that of the control group (3.0 ± 0.7) (*P* < 0.001), which fully indicates that this model can effectively stimulate students’ learning enthusiasm and improve their learning experience.

### 4.2 Practical value for the teaching reform of infectious diseases

#### 4.2.1 Practical value in different region

In the context of Chinese medical education, several studies have confirmed the effectiveness of SPOC combined with flipped classroom in improving students’ theoretical performance and clinical skills. Consistent with our findings, in similar projects implemented by institutions such as Kunming Medical University and Guangdong Medical University, students have demonstrated higher levels of learning satisfaction and teaching participation (27, 28). Although SPOC flipped classrooms have effectively promoted resource integration and student participation globally (29), the Chinese model emphasizes structured guidance and teacher feedback (such as optimizing educational quality by monitoring learning behaviors) (30). These differences reflect the influence of different educational and cultural traditions.

#### 4.2.2 Addressing core pain points in infectious diseases teaching

Infectious Diseases has the disciplinary characteristics of “rapid knowledge update, high practical requirements, and strong connection with public health.” Traditional LBL teaching is difficult to meet the development needs of the discipline due to the long update cycle of textbooks and weak practical links. The SPOC platform can update teaching resources in real-time. For example, in the chapter of “viral hepatitis,” the latest diagnosis and treatment plans and the characteristics of virus variants are added in a timely manner to solve the problem of “knowledge lag”; through practical links such as case discussions and epidemic prevention and control simulations, the flipped classroom strengthens students’ clinical diagnosis and treatment capabilities and public health emergency response capabilities, making up for the deficiency of “insufficient practice” (12). In addition, this model can also provide an objective basis for the evaluation of teaching quality through the learning data recorded by the platform, promoting the transformation of Infectious Diseases teaching from “experience-driven” to “data-driven” (11).

Notably, this model is highly relevant to post-COVID-19 medical education. After the pandemic, clinical medicine students faced challenges in transitioning from online-only to on-site teaching (31). The ‘online pre-class learning + offline interactive practice’ structure of the ‘SPOC + flipped classroom’ model effectively bridged this transition, as reflected in Table 2: 89% (54/61) of experimental group students reported ‘the model helped me adapt to on-site clinical learning’ (item under ‘Overall Teaching Effect’), confirming its value in post-pandemic education recovery.

#### 4.2.3 Connecting with the requirements of clinical post competence

Modern medical education emphasizes “post competence - oriented,” requiring medical students to have a solid theoretical foundation, proficient clinical skills, good communication skills, and team cooperation spirit. The teaching plans designed in this study for chapters such as “viral hepatitis” and “AIDS” allow students to be familiar with clinical diagnosis and treatment processes and public health practice scenarios in advance through real case analysis, doctor-patient communication simulations, and community prevention and control plan design, gradually equipping them with the core capabilities required of clinical doctors. In the long run, this teaching model can shorten the adaptation period of students from “campus learning” to “clinical work” and provide strong support for cultivating high-quality medical talents who meet the needs of clinical posts (32).

#### 4.2.4 Providing a replicable paradigm for the teaching reform of medical courses

The successful practice of the “SPOC + flipped classroom” model in Infectious Diseases teaching can provide a reference paradigm for other medical courses (such as Internal Medicine, Surgery, and Pediatrics). Its core experiences include: first, building a “hierarchical” teaching resource system to meet the learning needs of different students (29); second, designing “scenario-based” practical links to strengthen the ability of knowledge application (33); third, establishing a “closed-loop” teaching feedback mechanism to ensure the continuous improvement of teaching quality (34). In addition, the teacher training program and technical support process formed in this study can also provide practical guidance for other colleges and universities to carry out blended teaching, promoting the overall improvement of teaching quality in medical education.

### 4.3 Research limitations and future directions

#### 4.3.1 Research limitations

Firstly, the representativeness of the sample is limited. This study only selected students majoring in clinical medicine at Chongqing Medical University as the research objects, and the sample size of the experimental group (61 students) is relatively small. The extrapolation of the research results may be affected by factors such as region, college level, and major type. Therefore, it is necessary to conduct a larger-sample study in multi-center and multi-level medical colleges to further verify the results.

Secondly, the long-term effect has not been fully verified. This study only evaluated the teaching effect through the final examination and the satisfaction survey at the end of the course, and did not track the performance of students during clinical internships (such as internship assessment scores and evaluations from teaching teachers), so it is impossible to fully judge the impact of this model on students’ long-term clinical abilities.

Thirdly, the evaluation dimension was insufficiently comprehensive. The initial study relied mainly on quantitative data (academic performance, satisfaction questionnaires) and lacked in-depth qualitative exploration: ① For students, there was no structured interview to capture subjective experiences such as ‘adaptation difficulties to flipped classroom’ or ‘suggestions for resource optimization’; ② For teachers, only preliminary feedback was collected, without systematic investigation of their workload burden, teaching challenges, or improvement suggestions for the model. These gaps may lead to an incomplete understanding of the model’s practical application barriers.

Fourthly, future studies will address these limitations by: ① Conducting semi-structured interviews with 15–20 experimental group students (stratified by academic performance) to explore in-depth learning experiences, using thematic analysis to extract optimization directions; ② Designing a “Teacher Teaching Experience Questionnaire” (including 10 Likert-scale items and 3 open-ended questions) and conducting in-depth interviews with 8–10 participating teachers to analyze their workload, adaptation challenges, and training needs. This will help integrate both student and teacher perspectives to optimize the teaching model and improve its promotion feasibility.

#### 4.3.2 Future research directions

Firstly, expand the research scope and sample size. Conduct multi-center studies in collaboration with medical colleges and universities in different regions and at different levels, include students from different majors such as clinical medicine, preventive medicine, and nursing, to further verify the universality of the “SPOC + flipped classroom” model in Infectious Diseases teaching; at the same time, increase the sample size of the experimental group to improve the statistical validity of the research results. Secondly, carry out long-term follow-up studies. Establish a database of students’ learning and clinical practice, track and record the performance of students from course learning to clinical internship and even after employment, evaluate the impact of this model on students’ long-term career development, and provide a more comprehensive basis for the optimization of the teaching model. Thirdly, deepen the research on factors affecting teaching effectiveness. Use statistical methods such as multiple regression analysis and structural equation modeling to analyze the correlation between variables such as “students’ individual characteristics,” “teaching implementation elements,” and “platform technical support” and teaching effectiveness, identify key influencing factors, and optimize the teaching program in a targeted manner. Fourthly, optimize the functions of the SPOC platform. Combine artificial intelligence technology to develop a learning data analysis module to realize intelligent teaching support such as “automatic identification of students with learning difficulties-intelligent push of personalized resources-real-time prediction of learning effects,” and further improve the accuracy and efficiency of teaching (35).

#### 4.3.3 Feasibility in developing countries

Implementing this model in resource-limited developing countries requires consideration of four core dimensions: infrastructure, teacher training, cost-effectiveness, and sustainability. In terms of infrastructure, stable internet connections and basic computer equipment are essential. Teacher training involves investing time in developing their skills in online resource creation and interactive teaching. Regarding cost-effectiveness, although the initial investment is substantial, long-term costs can be reduced through resource sharing. For sustainability, it is recommended to start with small-scale pilots and gradually expand the implementation scope. Through international cooperation and open-source platforms (e.g., Moodle), even simplified versions of the SPOC + flipped classroom model can be implemented in resource-constrained environments.

## 5 Conclusion

This study confirmed through a randomized controlled trial that the “SPOC + flipped classroom” blended teaching model has significant advantages in Infectious Diseases teaching. Compared with the traditional LBL teaching model, this model can improve students’ academic performance through precise knowledge transfer, strengthen students’ clinical practical literacy through scenario-based ability training, and enhance students’ teaching satisfaction through active learning participation. The “three-stage closed-loop teaching process” (online independent learning before class-offline in-depth internalization in class - online expansion and consolidation after class), the hierarchical teaching resource system, and the all-round teaching implementation guarantee measures (resource guarantee, teacher training, and technical support) designed in the study have strong operability and replicability. They can provide a feasible practical path for the teaching reform of Infectious Diseases and a reference paradigm for blended teaching in other medical courses. In the future, it is necessary to further improve this model through multi-center and long-term follow-up studies, promote its wide application in the field of medical education, and help cultivate high-quality medical talents who meet the needs of the new era.

## Data Availability

The original contributions presented in this study are included in this article/Supplementary material, further inquiries can be directed to the corresponding authors.
