# Supplementary material for: SPOC (Small Private Online Course)-infused flipped classroom: a promising approach to address knowledge lag and practice gaps in infectious diseases teaching
Source: Front Med (Lausanne). 2025 Dec 16;12:1702681. doi: 10.3389/fmed.2025.1702681 (PMC12747948; doi:10.3389/fmed.2025.1702681)
Supplement: Supplementary file 1 [file Supplementary_file_1.docx]

# Teaching Satisfaction Questionnaire

# for Infectious Diseases

## Target Respondents: Five-Year Program Clinical Medicine Majors

## Survey Purpose: To compare and evaluate the application effects of different teaching models in the Infectious Diseases course and provide a basis for teaching optimization

## Survey Unit: Teaching and Research Office of Infectious Diseases, the Second Clinical College of Chongqing Medical University

## Survey Time: ______ Year ______ Month

### I. Letter to Respondents

Dear Students,

Hello! This questionnaire aims to understand the practical application effects of different teaching models in the *Infectious Diseases* course. Your true feedback is crucial for us to optimize teaching plans and improve teaching quality.

The questionnaire is completely anonymous, and there are no right or wrong answers. All data will only be used for this teaching research. We strictly adhere to the principle of privacy protection and will not disclose your personal information. Please fill in each question truthfully based on your actual experience during the course learning.

It is estimated that filling out the questionnaire will take 5-8 minutes. Thank you for your support and cooperation!

Teaching and Research Office of Infectious Diseases, the Second Clinical College of Chongqing Medical University

______ Year ______ Month

### II. Basic Information (Optional)

1. Which teaching model group do you belong to? □ SPOC + Flipped Classroom Group □ Traditional Lecture-Based Learning (LBL) Group
2. Grade: □ 2018 □ 2019 □ Others ______
3. Gender: □ Male □ Female

### III. Teaching Satisfaction Evaluation

#### Scoring Instructions

Please tick "√" under the corresponding score option based on your actual learning experience. The specific scoring criteria are as follows:

1 point = Very Dissatisfied | 2 points = Dissatisfied | 3 points = Average | 4 points = Satisfied | 5 points = Very Satisfied

| **Dimension** | No | Survey Content | 1 | 2 | 3 | 4 | 5 |
| --- | --- | --- | --- | --- | --- | --- | --- |
| 1. **Applicability of Teaching Resources (3 questions in total)** | 1 | The teaching resources provided in the course can clearly explain the core knowledge of Infectious Diseases |  |  |  |  |  |
|  | 2 | The teaching resources can help verify the mastery of knowledge points |  |  |  |  |  |
|  | 3 | The content of the teaching resources is highly consistent with the learning objectives of the core chapters |  |  |  |  |  |
| 1. **Effectiveness of Classroom Teaching (3 questions in total)** | 4 | The classroom teaching sessions can effectively solve the doubts encountered in learning |  |  |  |  |  |
|  | 5 | Classroom activities can help understand the connection between theoretical knowledge and clinical practice |  |  |  |  |  |
|  | 6 | The allocation of classroom time is reasonable and does not affect learning efficiency |  |  |  |  |  |
| 1. **After-Class Learning Support (3 questions in total)** | 7 | After-class tasks can help deepen the understanding of Infectious Diseases knowledge |  |  |  |  |  |
|  | 8 | Teachers' feedback on after-class tasks is timely and instructive |  |  |  |  |  |
|  | 9 | After-class learning assistance can help adjust the independent learning rhythm |  |  |  |  |  |
| 1. **Learning Initiative and Competence Improvement (3 questions in total)** | 10 | The current teaching model can stimulate your initiative to learn Infectious Diseases |  |  |  |  |  |
|  | 11 | Through the current teaching model, your mastery of the theoretical knowledge of Infectious Diseases is more solid |  |  |  |  |  |
|  | 12 | The current teaching model is helpful for improving your core competencies |  |  |  |  |  |
| 1. **Overall Teaching Effect (3 questions in total)** | 13 | The content of the core chapters covered in the course can meet the course learning objectives |  |  |  |  |  |
|  | 14 | The overall course arrangement is compatible with the current teaching model and can effectively achieve the course teaching objectives |  |  |  |  |  |
|  | 15 | On the whole, you recognize the application value of the current teaching model in Infectious Diseases teaching |  |  |  |  |  |

### IV. Open-Ended Questions

1. What do you think is the most prominent advantage of the current teaching model in the Infectious Diseases course?
2. In your opinion, what aspects of the current teaching model (such as resource content, classroom sessions, after-class support, etc.) need improvement during its implementation?
3. Compared with other teaching models you know (or based on your learning experience this time), what specific suggestions do you have for the subsequent teaching of Infectious Diseases?

### V. Questionnaire Instructions

1. Please ensure that all questions are answered. If you have no relevant experience with a certain question, you can choose "Average (3 points)" and provide additional explanations in the open-ended questions;
2. The more specific your answers to the open-ended questions are, the more helpful they will be for teaching optimization. It is recommended that you elaborate in detail;
3. After completing the questionnaire, you can submit it directly to the course teacher or through the designated online channel;
4. If you have any questions about the content of the questionnaire, you can contact the Teaching and Research Office of Infectious Diseases (Tel: 023-62887015; Email: crbxjys@cqmu.edu.cn).
